# Predicted risk factors associated with secondary infertility in women: univariate and multivariate logistic regression analyses

**DOI:** 10.3389/fmed.2023.1327568

**Published:** 2024-03-25

**Authors:** Wafa Fatima, Abdul Majeed Akhtar, Asif Hanif, Aima Gilani, Syed Muhammad Yousaf Farooq

**Affiliations:** ^1^Faculty of Allied Health Sciences, University Institute of Public Health, The University of Lahore, Lahore, Pakistan; ^2^Trauma and Orthopedic Surgery, Salford Royal NHS Foundation Trust Hospital, Manchester, United Kingdom; ^3^Faculty of Allied Health Sciences, University Institute of Radiological Sciences and Medical Imaging Technology, The University of Lahore, Lahore, Pakistan

**Keywords:** secondary infertility, logistic regression analysis, multivariate, univariate, artificial neural network (ANN)

## Abstract

**Introduction:**

Infertile women are those who regularly engage in unprotected intercourse for a period of at least 1 year and are unable to become clinically pregnant. Primary infertility means the inability of couples to conceive, without any previous successful pregnancies. Secondary Infertility refers to the inability to get pregnant for 12 months, after having a previous pregnancy for one time at least. The objectives of the current study were to analyze risk factors for secondary infertility and compare the predictive accuracy of artificial neural network (ANN) and multiple logistic regression models.

**Methods:**

The study was conducted at The University Institute of Public Health collecting data from Gilani Ultrasound Center 18 months after approval of synopsis. A total of 690 women (345 cases and 345 controls) were selected. The women selected for the case group had to be 20–45 years of age, had any parity, and had a confirmed diagnosis of secondary infertility.

**Results:**

Multiple logistic regression (MLR) and ANN were used. The chance of secondary infertility was 2.91 times higher in women living in a joint family [odds ratio (OR) = 2.91; 95% confidence interval (CI) (1.91, 4.44)] and was also 2.35 times higher for those women who had relationship difficulties with their husband [OR = 2.35; 95% CI (1.18, 4.70)]. Marriage at an earlier age was associated with secondary infertility with β being negative and OR being < 1 [OR = 0.94; 95% CI (0.88, 0.99)]. For the logistic regression model, the area under the receiver operative characteristic curve (ROC) was 0.852 and the artificial neural network was 0.87, which was better than logistic regression.

**Discussion:**

Identified risk factors of secondary infertility are mostly modifiable and can be prevented by managing these risk factors.

## 1 Introduction

Infertility as a disease affects a couple's social, psychological, economic, and sexual wellbeing and is a significant global health issue. Infertility is often defined as a couple's failure to conceive after between 12 and 24 months of unprotected intercourse. Primary infertility occurs when a couple has never been successful in conceiving, whereas secondary infertility occurs after a previously confirmed pregnancy (1). Infertility affects 10% to 15% of couples worldwide. In 35% and 45% of instances, respectively, the female and male variables are to blame, while the remaining couples either have a mix of factors or experience idiopathic infertility (2, 3). Different parts of the world have varied fertility rates, and 10–15% of married couples struggle to conceive. Primary infertility and secondary infertility are the two categories of infertility (4).

In 2010, almost 40 million couples actively sought infertility therapy, of which 34 million were in developing countries. Worldwide, there were approximately 48.5 (10–15%) million infertile couples. Women are thought to experience the highest rates of infertility (5). A significant majority of female factor infertility is due to the tubal factor. Pelvic inflammatory disease and acute salpingitis are the two most common causes of female factor infertility. Approximately 12% of pelvic infection episodes result in tubal damage, 23% after two episodes, and 54% after three episodes (6). Nevertheless, the reasons for infertility may vary geographically (7). When a woman is under 35 years of age, a fertility evaluation is often conducted after 1 year of regular unprotected sexual activity, and for those in the age group of 35 years or older, it is usually performed after 6 months. However, in women with irregular menstrual cycles or recognized risk factors for infertility, such as a history of pelvic inflammatory disease, endometriosis, or reproductive tract anomalies, the assessment may be initiated earlier (8).

Infertility in women can be diagnosed using various methods (9). Different risk factors are attributed to secondary infertility including lifestyle variables such as diet, obesity, drinking, smoking, and environmental hazards, as well as secondarily connected factors to human infertility such as childbirth complications, postpartum practices, and symptoms of sexually transmitted diseases (10). Other prevalent causes of female infertility include anovulatory disorders, polycystic ovarian syndrome, peri-tubovarian adhesions, endometriosis, and uterine and cervical factors (11, 12). Traditional statistical analysis approaches are used to discover the specific cause of infertility and give effective predictors of prevention and management. Univariate analysis can be used to assess the association between the investigated factor and the treatment result. However, multivariate analysis (multivariate logistic regression) provides a high-accuracy model for predicting pregnancy. Many types of studies utilize the phrase “data mining” to evaluate and classify medical data (13). There is great hope for artificial neural network (ANN) technology, which has already been shown to be successful in pregnancy prediction and can replace traditional statistical prediction methods, such as regression analysis. This classifier is designed to learn information, generalize, and model any linear or non-linear multidimensional accuracy (13).

Different statistical techniques are being used to explore determinants of medical conditions or classify them. The classification of individuals is a common problem, whereas the traditional statistical classification methods, such as logistic regression (LR), have been extensively used in the medical field to explore determinants when dependent variables had dichotomous outcomes (14, 15). MLR is taken from classical statistics based on probabilities and dominates the data, and it does not have the potential to solve non-linear problems (16).

## 2 Materials and methods

### 2.1 Study design and sampling

A case–control study was designed by non-probability consecutive sampling. This study was conducted at the University Institute of Public Health (UIPH), University of Lahore, Lahore, Pakistan, by incorporating data from the Gilani Ultrasound Center, Lahore, in a period of 18 months after approval of the synopsis. A total of 690 women (345 cases and 345 controls) were included in the analysis.

### 2.2 Inclusion and exclusion criteria

Inclusion criteria for the case group were women between the ages of 20 and 45 years, having any previous parity, and valid diagnosis of secondary infertility (as per operational definition). Inclusion criteria for controls were women between the ages of 20 and 45 years and having any parity. Exclusion criteria for cases and controls were couples that were separated for 1 year at least, couples with male factor infertility, and infertile women with a history of tuberculosis or any organic lesion (fibroids, etc.).

### 2.3 Ethical considerations

While conducting the study, the ethical guidelines established by the ethics council of the University of Lahore were followed, and the participants' rights were upheld. All participants provided their written and informed consent to participate in the study.

### 2.4 Data collection procedure

After receiving approval for the study protocol from the institutional review board (IRB) of the university, female patients with secondary infertility who fulfilled the inclusion and exclusion criteria were included in the study. The collection of data and all information were kept anonymous. All women underwent a complete physical examination, with measurement of height, weight, and body mass index (BMI). Demographical data such as age, years since marriage, and duration of infertility were collected. Complete medical history and examination were conducted and recorded. Information was recorded on *pro forma* and analyzed.

### 2.5 Data analysis

All data were recorded and analyzed using the Stata program. In descriptive analysis, for quantitative data, mean ± S.D was used, or in case of non-normality of data, median ± IQR was used. Independent sample *t*-test was applied for normally distributed data, and the Mann–Whitney *U*-test was applied for data that were not normally distributed. For categorical data, frequency (%) was used, and the chi-square test was used to analyze the significant association between cases and controls and other factors. In inferential statistics, multiple logistic regression was used in addition to ANN. The receiver operating characteristic (ROC) curve and the area under ROC were also calculated. The association was considered significant at a *P* ≤ 0.05.

## 3 Results

The mean ages of the women in the case group and control group were 33.08 ± 4.17 years and 31.37 ± 4.36 years, respectively. The median age was statistically higher in the case group (34.0 ± 6 years) than in the control group (30.0 ± 7 years), with a *P* < 0.05. Similar to age, other sociodemographic, anthropometric, and medical risk factors were studied in participants of both case and control groups. All significant variables or variables with *p* < 0.2 (number of variables = 20) were taken for logistic regression and artificial neural network analysis (Table 1). The final model was run by taking case and control as dependent variables, and independent variables were current age, age at marriage, obesity, working status, joint type of family, cousin marriage, relationship difficulties with husband, domestic violence during previous pregnancy by husband, H/O hypertension, H/O diabetes, H/O of polycystic ovary syndrome, H/O of pelvic inflammatory disease, H/O of endometriosis, H/O of uterine fibroids, menorrhagia, intermenstrual bleeding, BMI, H/O abortion, H/O of breastfeeding, and H/O urinary tract infection (UTI). Out of 20 predictors, 11 variables were selected in the final model, with R^2^ = 0.484, and the Hosmer–Lemeshow test was insignificant (*P* > 0.05) (Table 2).

**Table 1:** Variable risk factors with significant differences are taken for logistic regression and artificial neural network.

**Variable risk factors**		**Mean ±S.D**	**Median ±IQR**	***P*-value**
Current age	Study group	33.08 ± 4.17	34.0 ± 6	< 0.001^**^
	Control group	31.37 ± 4.36	30.0 ± 7	
	Total	32.22 ± 4.35	32.0 ± 6	
Age at marriage	Study group	24.33 ± 4.76	25.0 ± 6	0.003^*^
	Control group	24.59 ± 2.96	25.0 ± 5	
	Total	24.46 ± 3.96	25.0 ± 6	
BMI	Study group	27.61 ± 4.27	27.64 ± 7.56	< 0.001^**^
	Control group	25.52 ± 4.30	24.77 ± 6.73	
	Total	26.56 ± 4.41	25.86 ± 7.01	
		**Groups**		
		**Cases**	**Controls**	
Profession	Working	128 (37.1%)	97 (28.1%)	0.01^*^
	Housewife	217 (62.9%)	248 (71.9%)	
Obesity	Yes	104 (30.1%)	79 (22.9%)	0.03^*^
	No	241 (69.9%)	266 (77.1%)	
Family status	Combine	210 (60.9%)	128 (37.1%)	< 0.001^**^
	Nuclear	135 (39.1%)	217 (62.9%)	
Cousin marriage	Yes	224 (64.9%)	196 (56.8%)	0.03^*^
	No	121 (35.1%)	149 (43.2%)	
Relationship difficulties with husband	Yes	41 (11.9%)	19 (5.5%)	0.003^*^
	No	304 (88.1%)	326 (94.5%)	
Domestic violence by husband during previous pregnancy	Yes	50 (14.5%)	31 (9%)	0.03^*^
	No	295 (85.5%)	314 (91%)	
History of diabetes	Yes	55 (15.9%)	30 (8.7%)	0.004^*^
	No	290 (84.1%)	315 (91.3%)	
History of hypertension	Yes	90 (26.1%)	64 (18.6%)	0.02^*^
	No	255 (73.9%)	281 (81.4%)	
History of polycystic ovary syndrome	Yes	61 (17.7%)	33 (9.6%)	0.002^*^
	No	284 (82.3%)	312 (90.4%)	
History of pelvic inflammatory disease	Yes	25 (7.2%)	7 (2.0%)	0.001^*^
	No	320 (92.8%)	338 (98.0%)	
History of endometriosis	Yes	53 (15.4%)	32 (9.3%)	0.02^*^
	No	292 (84.6%)	313 (90.7%)	
Uterine fibroids	Yes	80 (23.2%)	56 (16.2%)	0.02^*^
	No	265 (76.8%)	289 (83.8%)	
Menorrhagia	Yes	53 (15.4%)	25 (7.2%)	0.001^*^
	No	292 (84.6%)	320 (92.8%)	
Intermenstrual bleeding	Yes	38 (11%)	20 (5.8%)	0.01^**^
	No	307 (89%)	325 (94.2%)	
History of abortion	Yes	172 (49.9%)	55 (15.9%)	< 0.001^**^
	No	173 (50.1%)	290 (84.1%)	
History of breastfeeding	Yes	162 (47%)	112 (32.5%)	< 0.001^**^
	No	183 (53%)	233 (67.5%)	
History of urinary tract infection	Yes	113 (32.8%)	37 (10.7%)	< 0.001^**^
	No	232 (67.2%)	308 (89.3%)	

^*^, ^**^Denote the severity of significant difference.

**Table 2:** Model summary and Hosmer–Lemeshow test.

**Model summary**			**Hosmer–Lemeshow test**		
**Step**	−**2 Log likelihood**	**R Square**	χ^2^	**df**	**Sig**.
1	863.348	0.168	0.000	0	.
2	813.307	0.250	7.610	2	0.022
3	782.025	0.298	34.008	4	0.000
4	733.996	0.368	49.408	8	0.000
5	710.596	0.400	17.420	8	0.026
6	689.261	0.428	21.845	8	0.005
7	672.821	0.450	19.601	8	0.012
8	661.632	0.464	28.816	8	0.000
9	655.558	0.471	40.104	8	0.000
10	650.709	0.477	59.845	8	0.000
11	645.224	0.484	12.176	8	0.144

**Figure 1:**
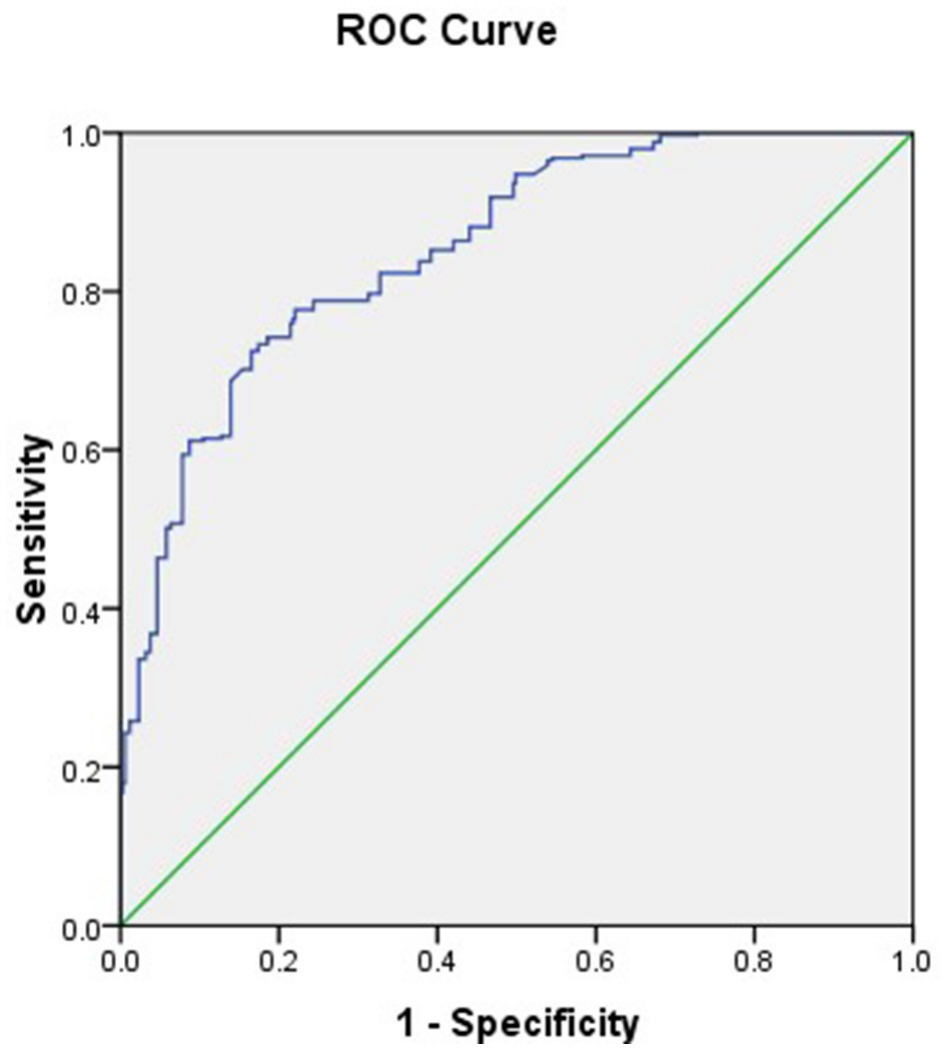
Receiver operating characteristic (ROC) of logistic regression model.

Advanced age had a positive impact on secondary infertility (β = 0.22), duration of marriage had a negative effect on secondary infertility (β = −0.07), working status had a positive effect on secondary infertility (β = 1.10), joint family had a positive effect on secondary infertility (β = 1.07), and cousin marriage and relationship difficulties with husband also had positive effect of secondary infertility as their βs were also positive, i.e., 0.58 and 0.86. H/O diabetes (β = 0.77), H/O of PID (β = 1.71), H/O abortion (β = 2.48), H/O of breastfeeding (β = 1.33), and H/O UTI (β = 1.79) all had positive effects on secondary infertility. The risk of secondary infertility in presence of H/O abortion was 11.98 [OR = 11.98; 95% CI (7.26, 19.76)] and was 5.98 times higher for H/O UTI [OR = 5.98; 95% CI (3.52, 10.19)], in presence of H/O of PID it had risk of 5.52 [OR = 5.52; 95% CI (1.64, 18.56)], H/O of breastfeeding was also responsible of secondary infertility as 3.79 times, i.e. [OR = 3.79; 95% CI (2.45, 5.87)]. Chances were three times higher to have secondary infertility for working women [OR = 3.00; 95% CI (1.90, 4.74)]. The risk of secondary infertility was 2.91 times higher in women living in a joint family [OR = 2.91; 95% CI (1.91, 4.44)] and was also 2.35 times higher for those women who had relationship difficulties with their husband [OR = 2.35; 95% CI (1.18, 4.70)]. Women had 2.15 times, 1.78 times, and 1.25 times higher chances of secondary infertility in the presence of H/O diabetes, cousin marriage, and current age, respectively, i.e. [OR = 2.15; 95% CI (1.14, 4.07)], [95% CI (1.17, 2.72)], [OR = 1.25; 95% CI (1.18, 1.32)]. Marriage at a young age was associated with secondary infertility as its β is negative and OR is < 1 [OR = 0.94; 95% CI (0.88, 0.99)] (Table 3). The final model was Logit (Y) = −8.51 +0.22^*^current age - 0.07^*^age at marriage + 1.10^*^working status + 1.07^*^joint family + 0.58^*^cousin marriage +0.86^*^ relationship difficulties with husband + 0.77^*^ H/O diabetes + 1.71^*^H/O of PID + 2.48^*^H/O abortion + 1.33^*^H/O of breastfeeding + 1.79^*^H/O UTI. For the logistic regression model, the area under ROC was 0.852 (95% CI: 0.825, 0.880) (Figure 1).

**Table 3:** Model of multiple logistic regression analysis.

	**β**	**S.E**	**Wald**	***P*-value**	**Adjusted OR**	**95.0% CI of OR**	
						**Lower**	**Upper**
Current age	0.22	0.03	55.33	0.00	1.25	1.18	1.32
Age at marriage	−0.07	0.03	5.50	0.02	0.94	0.88	0.99
Working status	1.10	0.23	22.21	0.00	3.00	1.90	4.74
Joint family	1.07	0.22	24.80	0.00	2.91	1.91	4.44
Cousin marriage	0.58	0.22	7.13	0.01	1.78	1.17	2.72
Relationship difficulties with husband	0.86	0.35	5.89	0.02	2.35	1.18	4.70
H/O diabetes	0.77	0.32	5.61	0.02	2.15	1.14	4.07
H/O of PID	1.71	0.62	7.61	0.01	5.52	1.64	18.56
H/O abortion	2.48	0.26	94.48	0.00	11.98	7.26	19.76
H/O of breastfeeding	1.33	0.22	35.96	0.00	3.79	2.45	5.87
H/O UTI	1.79	0.27	43.42	0.00	5.98	3.52	10.19
Constant	−8.51	0.99	73.34	0.00	0.00		

**Figure 2:**
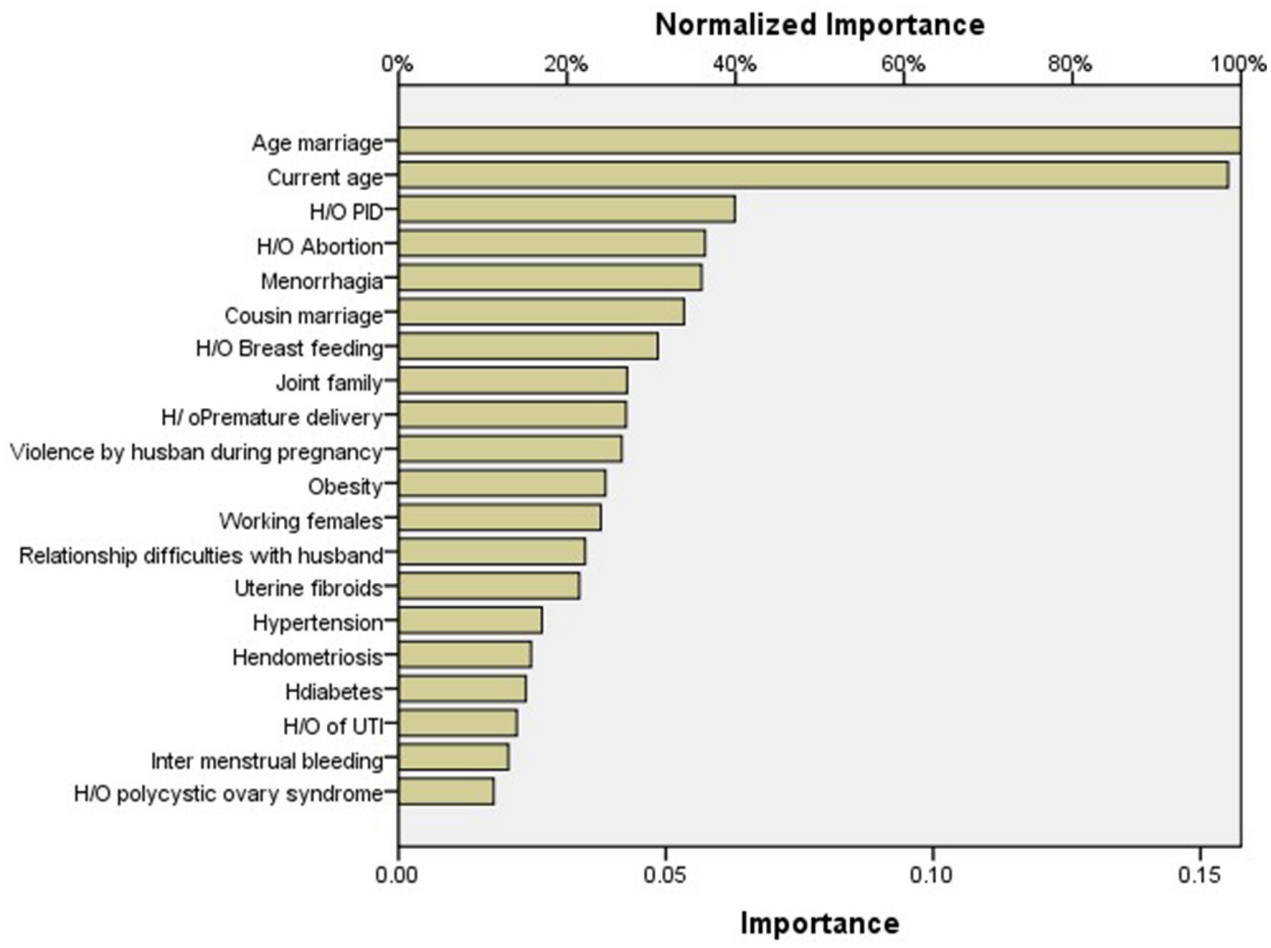
Normalized importance through artificial neural network.

**Figure 3:**
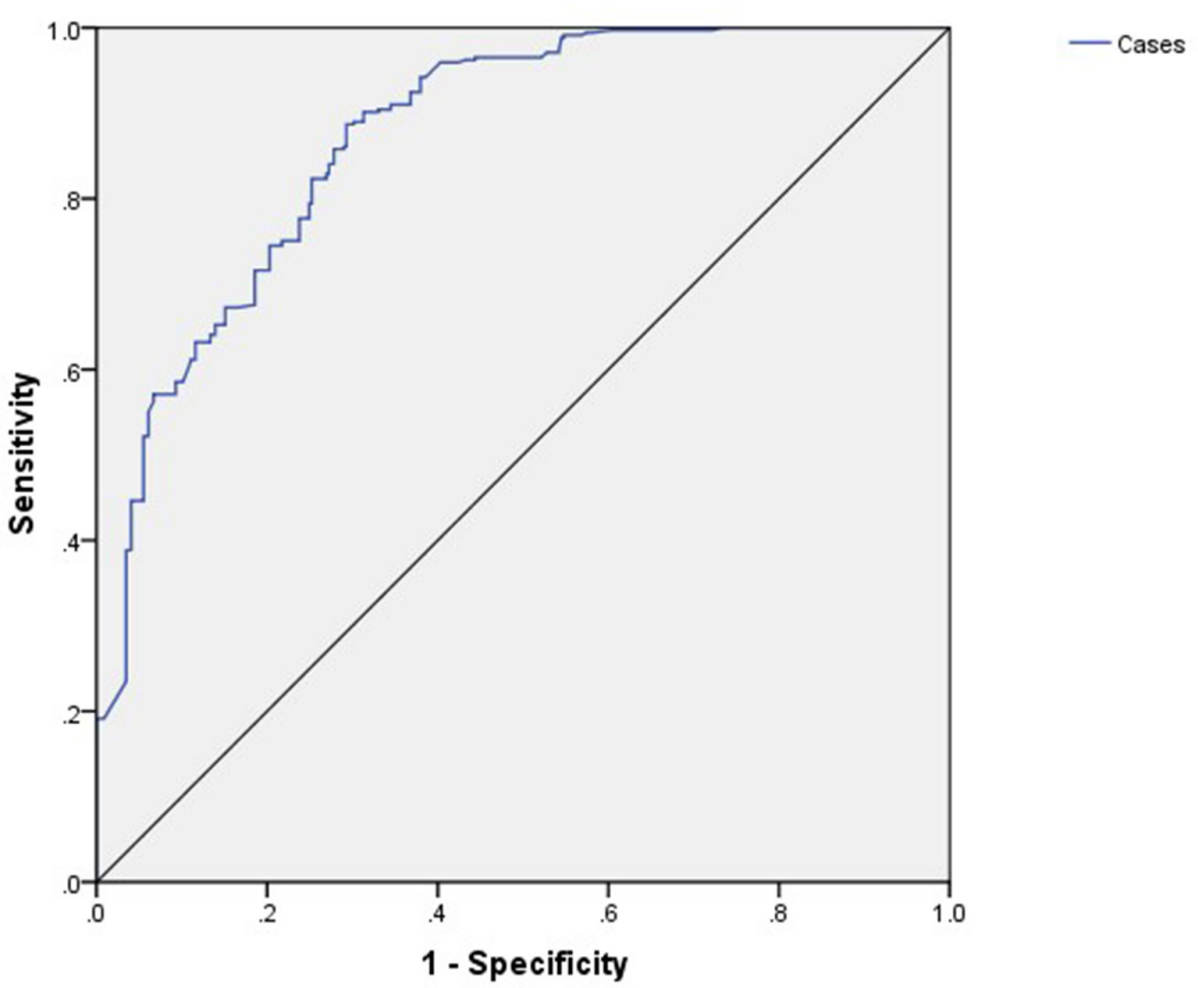
Receiver operating characteristic (ROC) for ANNs.

As per importance chart of artificial neural network, the highest normalized importance was given to age at marriage (100%), followed by current age (years) (98.5%), history of pelvic inflammatory disease (39.9%), history of abortion (36.4%), menorrhagia (36.0%), cousin marriage (33.9%), history of breastfeeding to child (30.8%), type of family (27.1%), premature delivery (27.0%), violence during previous pregnancy by husband (26.5%), obesity (24.6%), profession (24.0%), relationship difficulties with husband (22.2%), uterine fibroids (21.5%), history of hypertension (17.0%), history of endometriosis (15.7%), history of diabetes (15.1%), history of urinary tract infection (14.0%), history of polycystic ovary syndrome (11.3%), and intermenstrual bleeding (13.0%) (Table 4, Figure 2). For ANN, the area under the curve was 0.872, which was better than logistic regression (Figure 3).

**Table 4:** Normalized importance through artificial neural network.

	**Importance**	**Normalized importance**
Age at marriage (years)	0.158	100.0%
Current age (years)	0.155	98.5%
History of pelvic inflammatory disease	0.063	39.9%
History of abortion	0.057	36.4%
Menorrhagia	0.057	36.0%
Cousin marriage	0.053	33.9%
History of breastfeeding	0.048	30.8%
Type of family	0.043	27.1%
Premature delivery	0.043	27.0%
Violence during previous pregnancy by husband	0.042	26.5%
Obesity	0.039	24.6%
Profession	0.038	24.0%
Relationship difficulties with husband	0.035	22.2%
Uterine fibroids	0.034	21.5%
History of hypertension	0.027	17.0%
History of endometriosis	0.025	15.7%
History of diabetes	0.024	15.1%
History of urinary tract infection	0.022	14.0%
History of polycystic ovary syndrome	0.018	11.3%
Intermenstrual bleeding	0.021	13.0%

## 4 Discussion

The single most significant factor affecting both spontaneous and treatment-related conception is female age. The threshold for advanced reproductive age lacks a universally agreed-upon definition; however, it is generally acknowledged that 35 years marks a significant point in terms of fertility (17). In this study, patients who have secondary infertility had a mean age of 33.08 ± 4.17 years. A study conducted in 2010 by Nosheen et al. (18) reported that the mean age in secondary infertility was 32 years, while Talib et al. (19) reported in 2007 that the mean age of secondary infertility was 29.4 years in women. Factors related to nutrition and lifestyle that impact fertility encompass conditions, such as weight imbalances, anemia, and smoking. A report published by the American Society for Reproductive Medicine emphasizes that 12% of infertility instances arise from being either underweight or overweight. Additionally, a history of breastfeeding was associated with a higher likelihood of experiencing secondary infertility (20).

In the current study, ANNs gave better predictive accuracy based on their sensitivity and specificity and area under the curve (AUC). For the logistic regression model, the area under ROC was 0.852, and for the artificial neural network, the area under ROC was 0.87, which was better than logistic regression. While comparing techniques of machine learning, according to research, the most suitable fit point was found using the logistic regression ROC curve with a sensitivity of 0.688 and specificity of 0.615, and an ANN ROC curve with a sensitivity of 0.935 and specificity of 0.873 was obtained (21). Another study was conducted to predict the probability of preterm birth (PTB) using logistic regression, in which they reported that few variables significantly contributed to the risk of PTB. They found that there was an elevated probability of PTB for women under the age of 35 years, with an OR of 1.8 and a 95% confidence interval (1, 3), for refugees, with an OR of 1.57 and a 95% confidence interval (1.05, 2.34), for antenatal visits of at least four, with an OR of 2.89 and a 95% confidence interval (1.30, 6.4), for medically induced pregnancies, with an OR of 4.01 and a 95% confidence interval (1.30, 6.4), history of previous preterm delivery, with an OR of 5.58 and a 95% confidence interval (3.13, 9.94), for previous history of stillbirth, with an OR of 4.01 and a 95% confidence interval (1.59, 10.13), and for previous history of cesarean section, with an OR of 1.78 and a 95% confidence interval (1.00, 3.00) (22).

One recent publication in April 2019 determines the factors affecting birth weight by comparing the multiple logistic regression analysis and ANN. A total of 223 newborn babies in Istanbul, Turkey, were included in this study. The strategy designed based on these records was assessed using logistic regression and ANN. For the ANN and the logistic regression models, the area under the receiver operating characteristic (AuROC) curve was 0.941 (SD = 0.0012) and 0.909 (SD = 0.019), respectively, whereas the ANN value was greater than the LR value, and this investigation found that the outcomes were relatively similar (23). In the current study, for ANN, the area under the curve was 0.872, which was better than 165 logistic regressions.

Another study was conducted to compare logistic regression and ANNs to predict the outcomes in extremely low birth weight neonates. In that study, for both models, the AUC for analysis using the significant variables was greater than the AUC for analysis using the whole data set (*p* = 0.005). The AUC was mostly influenced by gestational age, birth weight, and the 5-min Apgar score, with similar contributions to the individual variables in both models. Based on significant variables at 80% sensitivity, specificity, PPV, and NPV were equal for both models (85% specificity, 72% PPV, and 90% NPV) (24). On the other side, the values of specificity and sensitivity for both groups were not the same.

In our study, among participants in the case group, 60.9% of women are living in a combined family system, 64.9% of women are married to their cousins, and 88.1% of women have relationship problems with their husbands, and these findings suggest that women should be very careful with respect to these factors before and after marriage. These risk factors emerged as potential risk factors that are associated with secondary infertility. Meanwhile, gynecological surgeries need to be considered carefully, especially surgeries on ovaries and fallopian tubes. Furthermore, women must stay away from possible factors that contribute to Decreased Ovarian Reserve (DOR), such as smoking and radiation, and live a healthy life.

## 5 Conclusion

This study identified that social and sociodemographic, anthropometric, family and social support, history of different medical illnesses, birth history, gynecological, and family history of different medical risk factors are the main causes of secondary infertility in women. Multiple logistic regression analysis was performed to check the specificity, sensitivity, and accuracy of the study. Hence, identified risk factors of secondary infertility are mostly modifiable and can be prevented or treated. By managing these risk factors, we can reduce the risk of secondary infertility.

## Data availability statement

The original contributions presented in the study are included in the article/supplementary material, further inquiries can be directed to the corresponding author.

## Ethics statement

The studies involving humans were approved by University of Lahore's ethics council. The studies were conducted in accordance with the local legislation and institutional requirements. The participants provided their written informed consent to participate in this study.

## Author contributions

WF: Conceptualization, Data curation, Investigation, Methodology, Writing—original draft. AMA: Data curation, Writing—original draft. AH: Formal analysis, Methodology, Writing—review & editing. AG: Methodology, Writing—review & editing. SF: Formal analysis, Writing—review & editing.
